# Mechanical power is associated with weaning outcome in critically ill mechanically ventilated patients

**DOI:** 10.1038/s41598-022-21609-2

**Published:** 2022-11-16

**Authors:** Yao Yan, Yongpeng Xie, Xiaobing Chen, Yan Sun, Zhiqiang Du, Yanli Wang, Xiaomin Li

**Affiliations:** 1 Department of Emergency Medicine, Lianyungang Clinical College of Nanjing Medical University, The First People’s Hospital of Lianyungang City, Lianyungang, 222000 Jiangsu China; 2 Department of Critical Care Medicine, The Second People’s Hospital of Lianyungang City, Lianyungang, 222000 Jiangsu China

**Keywords:** Respiratory distress syndrome, Prognostic markers

## Abstract

Several single-center studies have evaluated the predictive performance of mechanical power (MP) on weaning outcomes in prolonged invasive mechanical ventilation (IMV) patients. The relationship between MP and weaning outcomes in all IMV patients has rarely been studied. A retrospective study was conducted on MIMIC-IV patients with IMV for more than 24 h to investigate the correlation between MP and weaning outcome using logistic regression model and subgroup analysis. The discriminative ability of MP, MP normalized to dynamic lung compliance (C_dyn_-MP) and MP normalized to predicted body weight (PBW-MP) on weaning outcome were evaluated by analyzing the area under the receiver-operating characteristic (AUROC). Following adjustment for confounding factors, compared with the reference group, the Odds Ratio of weaning failure in the maximum MP, C_dyn_-MP, and PBW-MP groups increased to 3.33 [95%CI (2.04–4.53), *P* < 0.001], 3.58 [95%CI (2.27–5.56), *P* < 0.001] and 5.15 [95%CI (3.58–7.41), *P* < 0.001], respectively. The discriminative abilities of C_dyn_-MP (AUROC 0.760 [95%CI 0.745–0.776]) and PBW-MP (AUROC 0.761 [95%CI 0.744–0.779]) were higher than MP (AUROC 0.745 [95%CI 0.730–0.761]) (*P* < 0.05). MP is associated with weaning outcomes in IMV patients and is an independent predictor of the risk of weaning failure. C_dyn_-MP and PBW-MP showed higher ability in weaning failure prediction than MP.

## Introduction

Mechanical ventilation is an important life-saving support measure for Intensive Care Unit (ICU) patients; however, prolonged mechanical ventilation may lead to complications such as pneumonia, barotrauma, diaphragmatic insufficiency and ventilator atrophy^1^. Simultaneously, delays in weaning are associated with increased risk of morbidity, mortality and prolonged hospital stay^2^. Therefore, accurate assessment of weaning timing, following etiology improvement is of paramount importance in the management of patients on invasive mechanical ventilation. Intensive care professionals are expected to find a fine balance between delayed and premature weaning to avoid increased risks of ventilator-related complications, extubation failure and artificial airway reconstruction^3^.

Mechanical power (MP) is the energy delivered by the ventilator to the entire respiratory system per time unit and combines all factors affecting the energy load of the respiratory system, including pressure, tidal volume, flow rate and respiratory rate^4^. MP is primarily calculated as the product of the applied airway pressure and minute ventilation and can be used as an estimate of the workload exerted on the respiratory muscles during spontaneous breathing^5^. MP is a major determinant to ensure adequate gas exchange in the body and a key factor in assessing the ability of a patient to successfully wean from mechanical ventilation^6^.

Recent studies concluded that MP and MP normalized to dynamic lung compliance (C_dyn_-MP) before the first spontaneous breathing trial (SBT) were independently associated with weaning outcomes in tracheotomized patients on prolonged mechanical ventilation and were identification markers for weaning failure in high-risk patients^5–7^. However, the number of tracheotomized patients involved in the study was low, came from a single center and no patients with tracheal intubation were included. Therefore, a larger scale clinical study is needed to further verify the relationship between MP and weaning outcomes in all critically ill mechanically ventilated patients. The present study aims to investigate the association between MP and weaning outcomes in mechanically ventilated patients based on the MIMIC-IV v1.0 database.

## Methods

This is a retrospective study based on the Medical Information Mart for Intensive Care IV v1.0 (MIMIC-IV), which contains comprehensive, high-quality data on 76,540 ICU admissions at Beth Israel Deaconess Medical Center in Boston (BIDMC), Massachusetts, from 2008 to 2019. The researcher (YY) completed the online course "Protecting Human Research Participants" offered by the National Institutes of Health, gained access to the database and was responsible for data extraction (Certification Number: 41699414). This study was an analysis of a third-party anonymous public database. All methods were carried out in accordance with the relevant guidelines and regulations. The database was approved by the BIDMC and the Massachusetts Institute of Technology (MIT) Institutional Review Board (IRB) with an informed consent waiver.

### Patient selection

All patients in the MIMIC-IV database were screened and the following inclusion criteria were used: (i) age ≥ 18 years at the time of ICU admission; (ii) invasive mechanical ventilation time of at least 24 h; (iii) first time ICU admission. Exclusion criteria included: (i) lack of SBT records, non-first SBT records at ICU admission, and non-first extubation records; (ii) patients with incomplete ventilation data required to calculate MP; (iii) patients without T-tube test for SBT weaning. T-tube test refers to a trial of spontaneous breathing where the patient was disconnected from the ventilator and allowed to breathe spontaneously through a T-tube circuit for up to 30 min or up to 120 min with the FiO_2_ set at the same level as that used during mechanical ventilation. The patients were divided into weaning success group and weaning failure group, according to their weaning outcomes. Further division according to the time from the first SBT to extubation was performed, with the weaning failure group divided into group 1 (≤ 24 h), group 2 (2–7 days), and group 3 (> 7 days).

### Data collection

Data was extracted from the database using SQL using Navicat Premium version 15.0.9 (PremiumSoft, Navicat, CN). Age, gender, body mass index (BMI), smoking history, simplified acute physiology score II (SAPS II), sequential organ failure assessment (SOFA), sources of admission, comorbidities, intubation time, mechanical ventilation start time, start and end time of the first SBT, end time of mechanical ventilation, time of first extubation and second intubation, and time of the first non-invasive ventilation after extubation were recorded. Comorbidities such as hypertension, diabetes, chronic obstructive pulmonary disease (COPD), congestive heart failure, coronary artery disease, chronic kidney disease, and stroke were collected according to the ICD-9 codes recorded in the MIMIC-IV database. The average value of each 4-h time frame of respiratory mechanics parameters within 24 h before the first SBT was recorded, including tidal volume (V_T_), respiratory rate (RR), peak inspiratory pressure (P_peak_), plateau pressure (P_plat_), positive end expiratory pressure (PEEP) and minute ventilation (MV). The patient’s rapid shallow breathing index (RSBI) was recorded at the bedside before the first SBT. RSBI was calculated by dividing respiratory rate by tidal volume, that is RSBI = RR /V_T_. The mean values of physiological parameters, including vital signs and arterial blood gas analysis, were recorded during SBT. At the same time, laboratory indicators such as white blood cell count (WBC), platelets (PLT), hemoglobin (Hb), albumin, serum creatinine (SCr) and Urine output rate (Uorate) before the start of SBT were recorded.

### Calculation of MP

Total respiratory rate (RR) in MIMIC-IV database was used for current analysis. Positive end-expiratory pressure (PEEP) as recorded was the external or applied PEEP, not the total PEEP, or intrinsic PEEP. The plateau pressure (P_plat_) was measured during an inspiratory pause on the ventilator. Peak inspiratory pressure (P_peak_) should be obtained while the patient is relaxed, not coughing or moving in bed. MP was calculated according to Gattinoni's simplified mechanical power equation as follows^4^:

$${\text{MP}}\left( {{\text{J}}/{\text{min}}} \right) = 0.0{98} \times {\text{V}}_{{\text{T}}} \times {\text{RR}} \times ({\text{P}}_{{{\text{peak}}}} - 0.{5} \times \Delta {\text{P}}).$$

Driving pressure (∆P) in the ventilation mode was calculated using P_plat_ and PEEP:

$$\Delta {\text{P }}\left( {{\text{cmH}}_{{2}} {\text{O}}} \right) = {\text{P}}_{{{\text{plat}}}} - {\text{PEEP}}.$$

Dynamic lung compliance (C_dyn_) refers to the change in lung volume caused by a unit pressure change, reflecting the compliance of the overall respiratory system^7^ and was calculated using V_T_ and ∆P_aw_:

$${\text{C}}_{{{\text{dyn}}}} = {\text{ V}}_{{\text{T}}}/\Delta {\text{P}}_{{{\text{aw}}}} .$$

Dynamic driving pressure (∆P_aw_) in the ventilation mode was calculated using P_peak_ and PEEP^8^:

$$\Delta {\text{P}}_{{{\text{aw}}}} \left( {{\text{cmH}}_{{2}} {\text{O}}} \right) \, = {\text{P}}_{{{\text{peak}}}} - {\text{ PEEP}}.$$

MP normalized to dynamic lung compliance (C_dyn_-MP) was calculated using MP and C_dyn_^3^:

$${\text{C}}_{{{\text{dyn}}}}{\text{-MP }}\left( {{\text{J}}/{\text{min}} \times {\text{cmH}}_{{2}} {\text{O}}/{\text{ml}} \times {1}0^{{ - {3}}} } \right) = {\text{ MP }}/{\text{C}}_{{{\text{dyn}}}} .$$

MP normalized to predicted body weight (PBW-MP) was calculated using MP and predicted body weight (PBW)^9^:

$${\text{PBW-MP }}\left( {{\text{J}}/{\text{min}}/{\text{kg}}} \right) = {\text{ MP}}/{\text{PBW}}.$$

Calculation of PBW^9^:

$${\text{PBW }}\left( {{\text{male}}} \right) = { 5}0 + 0.{91 }\left[ {{\text{height }}\left( {{\text{cm}}} \right) - { 152}.{4}} \right],$$

$${\text{PBW }}\left( {{\text{female}}} \right) = { 45}.{5 } + 0.{91 }\left[ {{\text{height }}\left( {{\text{cm}}} \right) -{ 152}.{4}} \right].$$

In the study, C_dyn_-MP and PBW-MP are collectively referred to as normalized MP (norMP).

### Definitions and outcomes

SBT test refers to the use of T-tube or low-level pressure support in the spontaneous breathing mode in patients receiving invasive mechanical ventilation. The SBT test is performed to evaluate the ability of the patient to fully tolerate spontaneous breathing, and to assess whether the machine can be weaned, through a short-term dynamic observation^10^. Only patients on T-tube ventilation during weaning were included in this study to reduce the influence and bias of different SBT modalities on weaning outcomes. Weaning failure was defined as a patient who did not pass SBT or required re-intubation or non-invasive ventilation within 48 h of cessation of mechanical ventilation or died within 48 h of extubation^11^. In MIMIC-IV database, premature termination of SBT was assigned as follows: respiratory rate > 35 beats/min > 5 min; heart rate > 140 beats/min; blood pressure > 180 or < 90 mmHg; new-onset arrhythmia; pulse oximetry (SpO_2_) < 90% > 2 min; with use of accessory respiratory muscles. SBT was discontinued when the clinicians at the bedside observed that the patient's vital signs exceed the above indicators. The primary outcome of this process was the grouping of weaning success and failure patients, and secondary outcomes included duration of ventilation, length of stay in ICU, length of stay in hospital, 28-day re-intubation rate, 28-day mortality, and 28-day ventilator-free days.

### Statistical analysis

The normally distributed continuous variables were expressed as mean ± standard deviation and compared using Student’s t-test. Variables that were not normally distributed were expressed as median (interquartile range, IQR) and compared using the Mann–Whitney U test or the Kruskal–Wallis H test. The categorical variables were expressed as numbers (percentages) and compared using the chi-square test. The calculations for ∆P, ∆P_aw_, C_dyn_, MP, C_dyn_-MP, PBW-MP were performed according to the parameters collected every 4 h time frame 24 h before the start of the first SBT. The respiratory mechanics parameters closest to weaning (i.e., the last 4 h time frame 24 h before SBT) were used for comparisons between the weaning success group and the weaning failure group. The possible confounding factors were identified through univariate logistic regression analysis, and MP, C_dyn_-MP, and PBW-MP were included as study variables into logistic regression models A, B, and C, respectively. The relationship between study variables and weaning outcomes was verified after adjusting for confounding factors. The results are expressed as odds ratio (OR) and 95% confidence interval (95% CI). Taking the normal range of MP, C_dyn_-MP, and PBW-MP as the reference group, the logistic regression models of six subgroups of each variable were established, and the fitting curves were drawn to evaluate the correlation between C_dyn_, MP, C_dyn_-MP, PBW-MP and relative weaning failure risk (OR value). The variance inflation factor (VIF) was employed to detect potential multicollinearity in the model, with VIF ≥ 5 indicating the existence of multicollinearity. Receiver operating characteristic curves (ROC curves) were used to assess the discriminative power of the selected variables for weaning outcomes, and the diagnostic accuracy was expressed as the area under the receiver operating characteristic (AUROC). All tests were two-tailed, and *P* < 0.05 was considered statistically significant. Data analysis was performed using Stata V16.0 (StataCorp LLC, Texas, USA) software and graphs were designed in GraphPad Prism 8.0 (GraphPad Prism Software).

### Ethics approval and consent to participate

This study was an analysis of a third-party anonymous public database. The database was approved by the Beth Israel Deaconess Medical Center (BIDMC) and the Massachusetts Institute of Technology (MIT) Institutional Review Board (IRB) with an informed consent waiver.

## Results

**Figure 1:**
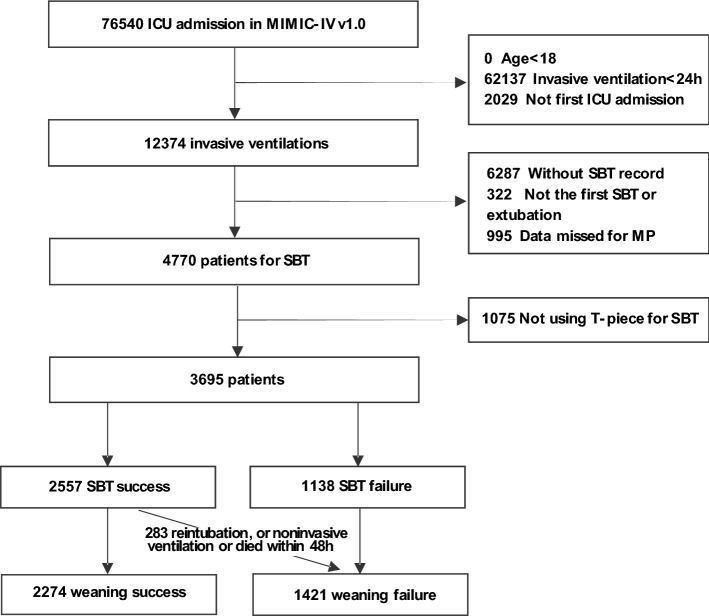
Flow chart of the study.

The MIMIC-IV database contains data from 76,540 patients admitted to the ICU from 2008 to 2019. A total of 3,695 patients who underwent invasive mechanical ventilation for more than 24 h and had a complete first SBT record were included in the study, according to the inclusion criteria established previously. The research flow chart is shown in Fig. 1. The average age of all patients was 66.8 (55.4–77.5) years old, 2102 (56.9%) were male, and the failure rate of weaning after the first SBT was 38.5% (1421/3695). The incidence of re-intubation, non-invasive mechanical ventilation or death within 48 h after the first successful SBT was 11.1% (283/2557). Significant differences were detected in BMI, SAPS II, SOFA, source from surgical intensive care unit (SICU), WBC, albumin, SCr, Uorate and physiological parameters during weaning between the successful weaning group and the failed weaning group (Table 1). Except for tidal volume, significant differences were observed in ventilation variables, MP, and norMP between the successful and failed weaning groups (Table 1). All prognostic indicators were significantly different between the two groups of patients (Table 2).

**Table 1:** Comparison of baseline characteristics between weaning success and weaning failure.

Variables	All	Weaning success	Weaning failure	*p* value
	(n = 3695)	(n = 2274)	(n = 1421)	
Age (years)	66.8 (55.4–77.5)	67.3 (55.7–77.9)	66.5 (54.6–76.4)	0.052
Gender (male)	2102 (56.9)	1275 (56.1)	827 (58.2)	0.203
BMI (kg/m^2^)	28.0 (24.2–33.3)	27.6 (24.1–32.5)	28.5 (24.5–34.0)	< 0.001
Smoking history	315 (8.5)	187 (8.2)	128 (9.0)	0.406
SAPS II	43 (34–53)	42 (33–52)	44 (36–55)	< 0.001
SOFA	7 (4–10)	6 (4–9)	8 (5–11)	< 0.001
**Sources of admission**				
MICU	871 (23.6)	546 (24.0)	325 (22.9)	0.427
SICU	609 (16.5)	404 (17.8)	205 (14.4)	0.008
TSICU	620 (16.8)	371 (16.3)	249 (17.5)	0.339
MICU/SICU	547 (14.8)	328 (14.4)	219 (15.4)	0.411
CVICU	465 (12.6)	267 (11.7)	198 (13.9)	0.051
CCU	359 (9.7)	216 (9.5)	143 (10.1)	0.573
NSICU	203 (5.5)	129 (5.7)	74 (5.2)	0.546
Others	21 (0.6)	13 (0.6)	8 (0.6)	0.973
**Comorbidities**				
Hypertension	1479 (40.0)	902 (39.7)	577 (40.6)	0.571
Diabetes mellitus	1138 (30.8)	685 (30.1)	453 (31.9)	0.261
COPD	256 (6.9)	157 (6.9)	99 (7.0)	0.942
Congestive heart failure	1107 (30.0)	675 (30.0)	432 (30.4)	0.643
Coronary artery disease	1015 (27.5)	620 (27.3)	395 (27.8)	0.724
Chronic kidney disease	838 (22.7)	513 (22.6)	325 (22.9)	0.826
Stroke	733 (19.8)	439 (19.3)	294 (20.7)	0.305
**Parameters before SBT 4 h**				
V_T_ (ml)	451 (395–512)	452 (392–519)	451 (398–507)	0.498
PEEP (cmH_2_O)	5.0 (5.0–8.0)	5.0 (5.0–5.3)	6.0 (5.0–10.0)	< 0.001
P_plat_ (cmH_2_O)	17.5 (15.0–20.5)	17.0 (14.0–20.0)	19.0 (16.0–22.0)	< 0.001
ΔP (cmH_2_O)	11.3 (9.0–14.0)	11.5 (9.0–14.0)	11.0 (9.0–14.0)	0.271
P_peak_ (cmH_2_O)	19.0 (15.0–24.0)	17.0 (12.8–21.0)	23.0 (19.0–27.0)	< 0.001
ΔP_aw_ (cmH_2_O)	13.0 (9.4–16.5)	11.7 (8.0–15.5)	15.0 (12.0–18.0)	< 0.001
RR (bpm)	19 (16–22)	18 (16–22)	20 (17–24)	< 0.001
MV (l/min)	8.2 (6.9–9.9)	7.9 (6.7–9.5)	8.8 (7.3–10.6)	< 0.001
FiO_2_ (%)	40 (40–50)	40 (40–50)	40 (40–50)	< 0.001
MP (J/min)	11.1 (7.4–16.2)	9.2 (6.0–13.2)	14.6 (10.6–20.2)	< 0.001
C_dyn_ (ml/cmH_2_O)	33.9 (26.2–49.2)	39.0 (28.5–62.0)	29.6 (24.2–36.7)	< 0.001
C_dyn_-MP (J/min × cmH_2_O/ml × 10^–3^)	331.5 (163.9–563.6)	231.5 (101.7–421.6)	501.3 (323.4–744.8)	< 0.001
PBW-MP (J/min/kg)	0.1881 (0.1234–0.2696)	0.1520 (0.0995–0.2200)	0.2452 (0.1837–0.3331)	< 0.001
RSBI (bpm/L)	41.7 (32.6–53.7)	40.2 (31.0–51.7)	44.3 (35.1–56.5)	< 0.001
**Laboratory data at the start of SBT**				
WBC (k/μl)	11.5 (8.5–15.8)	11.2 (8.3–15.3)	11.9 (8.8–16.9)	< 0.001
PLT (k/μl)	163 (109–228)	162 (111–224)	164 (108–233)	0.502
Hb (g/dl)	10.0 (8.6–11.5)	9.9 (8.6–11.4)	10.0 (8.6–11.6)	0.282
Albumiun (g/dl)	2.9 (2.5–3.4)	3.0 (2.6–3.4)	2.9 (2.4–3.4)	0.013
SCr (mg/dl)	1.1 (0.7–1.8)	1.0 (0.7–1.7)	1.2 (0.8–2.0)	< 0.001
Uorate before SBT (ml/kg/h)	0.6 (0.4–1.1)	0.7 (0.4–1.2)	0.6 (0.3–1.1)	< 0.001
**Physiological variables during SBT**				
HR (bpm)	83 (72–97)	82 (71–95)	86 (73–99)	< 0.001
BF (bpm)	19 (16–22)	18 (15–21)	20 (17–24)	< 0.001
MBP (mmHg)	75 (68–84)	76 (68–85)	74 (67–83)	< 0.001
SPO_2_ (%)	98 (97–100)	99 (97–100)	98 (96–100)	< 0.001
Temperature (℃)	37.1 (36.8–37.5)	37.1 (36.8–37.4)	37.1 (36.8–37.6)	0.024
PH (mmHg)	7.39 (7.34–7.44)	7.40 (7.35–7.44)	7.38 (7.33–7.43)	< 0.001
PaO_2_ (mmHg)	105 (84–130)	106 (86–132)	105 (83–129)	0.331
PaCO_2_ (mmHg)	39 (34–45)	39 (34–44)	39 (34–45)	0.926
PF (mmHg)	240 (180–323)	253 (193–333)	226 (163–305)	< 0.001

Data are median (interquartile range) or no./total (%).

BMI, body mass index; SAPS II, simplified acute physiology score II; SOFA, sequential organ failure assessment; MICU, Medical Intensive Care Unit; SICU, Surgical Intensive Care Unit; TSICU, Trauma SICU; MICU/SICU, Medical/ Surgical Intensive Care Unit; CVICU, Cardiac Vascular Intensive Care Unit; CCU, Coronary Care Unit; NSICU, Neuro Surgical Intensive Care Unit; COPD, chronic obstructive pulmonary disease; SBT, spontaneous breathing trial; V_T_, tidal volume; PEEP, positive end expiratory pressure; P_plat_, plateau pressure; ΔP, driving pressure; P_peak_, peak inspiratory pressure; ΔP_aw_, dynamic driving pressure (defined as P_peak_ – PEEP); RR, respiratory rate; MV, minute ventilation; MP, mechanical power; C_dyn_, dynamic lung compliance; C_dyn_-MP, MP normalized to dynamic lung compliance; PBW-MP, MP normalized to predicted body weight; RSBI, rapid shallow breathing index; WBC, white blood cell; PLT, platelet count; Hb, hemoglobin; SCr, serum creatinine; Uorate, Urine output rate; HR, heart rate; BF, breathing frequency; MBP, mean blood pressure; SPO_2_, pulse oximetry; PF, arterial partial pressure of oxygen (PaO_2_) divided by the inspired oxygen concentration (FiO_2_).

**Table 2:** Main outcomes in the weaning success and weaning failure groups. Data are median (interquartile range) or no./total (%). LOS, length of stay.

Weaning outcome	All	Weaning success	Weaning failure	*p* value
	(n = 3695)	(n = 2274)	(n = 1421)	
Weaning success	2274 (61.5)	2274 (100.0)	–	–
Weaning failure	1421 (38.5)	–	1421 (100.0)	–
Duration of ventilation (days)	3.7 (1.8–8.1)	2.5 (1.5–4.8)	7.3 (4.0–11.8)	< 0.001
ICU LOS (days)	6.8 (4.0–11.8)	5.2 (3.4–8.6)	10.2 (6.6–15.6)	< 0.001
Hospital LOS (days)	14.8 (9.0–23.3)	13.0 (8.3–21.0)	17.8 (11.2–27.0)	< 0.001
28-day reintubation	531 (14.4)	118 (5.2)	413 (29.1)	< 0.001
28-day mortality	693 (18.8)	282 (12.4)	411 (28.9)	< 0.001
28-day ventilator-free days	23.2 (14.3–26.0)	25.3 (21.5–26.5)	17.2 (1.5–22.6)	< 0.001

**Figure 2:**
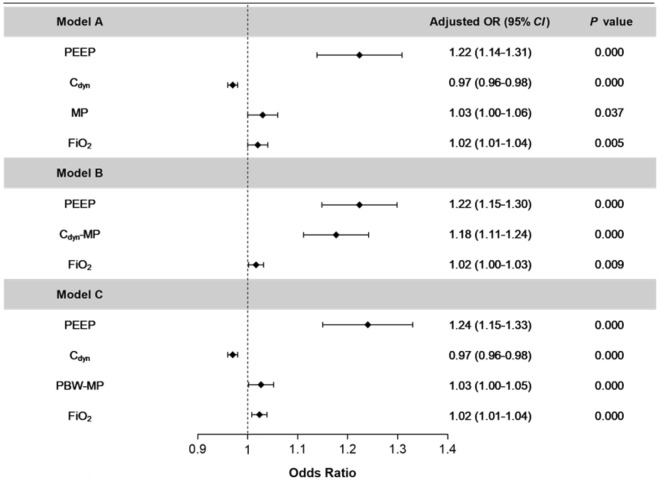
Forest plot of odds ratios for weaning failure to the population average after adjustment for the concomitant variable (age, BMI, SOFA, sources of admission (SICU), PEEP, P_plat_, ΔP_aw_, RR, MV, C_dyn_, FiO_2_ and HR, MBP, SPO_2_, PH, PF during SBT, and WBC, albumin, SCr, uorate at the start of SBT), respectively. Model A: MP as included in the regression model as an independent variable. Model B: C_dyn_-MP was included in the regression model as an independent variable. Model C: PBW-MP was included in the regression model as an independent variable. PEEP: positive end expiratory pressure; C_dyn_: dynamic lung compliance; MP: mechanical power; FiO_2_: inspired oxygen concentration; C_dyn_-MP: MP normalized to dynamic lung compliance; PBW-MP: MP normalized to predicted body weight.

Results of univariate logistic regression analyses related to weaning outcomes are presented in Supplementary Table S2. After adjustment for confounders, MP (OR 1.03 [95%CI (1.00–1.06), *P* = 0.037]), C_dyn_-MP (OR 1.18 per 100 J/min × cmH_2_O/ml × 10^–3^ [95%CI (1.11–1.24), *P* < 0.001]), PBW-MP (OR 1.03 per 0.01 J/min/kg [95%CI (1.00–1.05), *P* < 0.001]) were independently associated with the outcome of weaning failure from three independent logistic regression models A, B, and C for ICU patients with invasive mechanical ventilation (Fig. 2).

**Figure 3:**
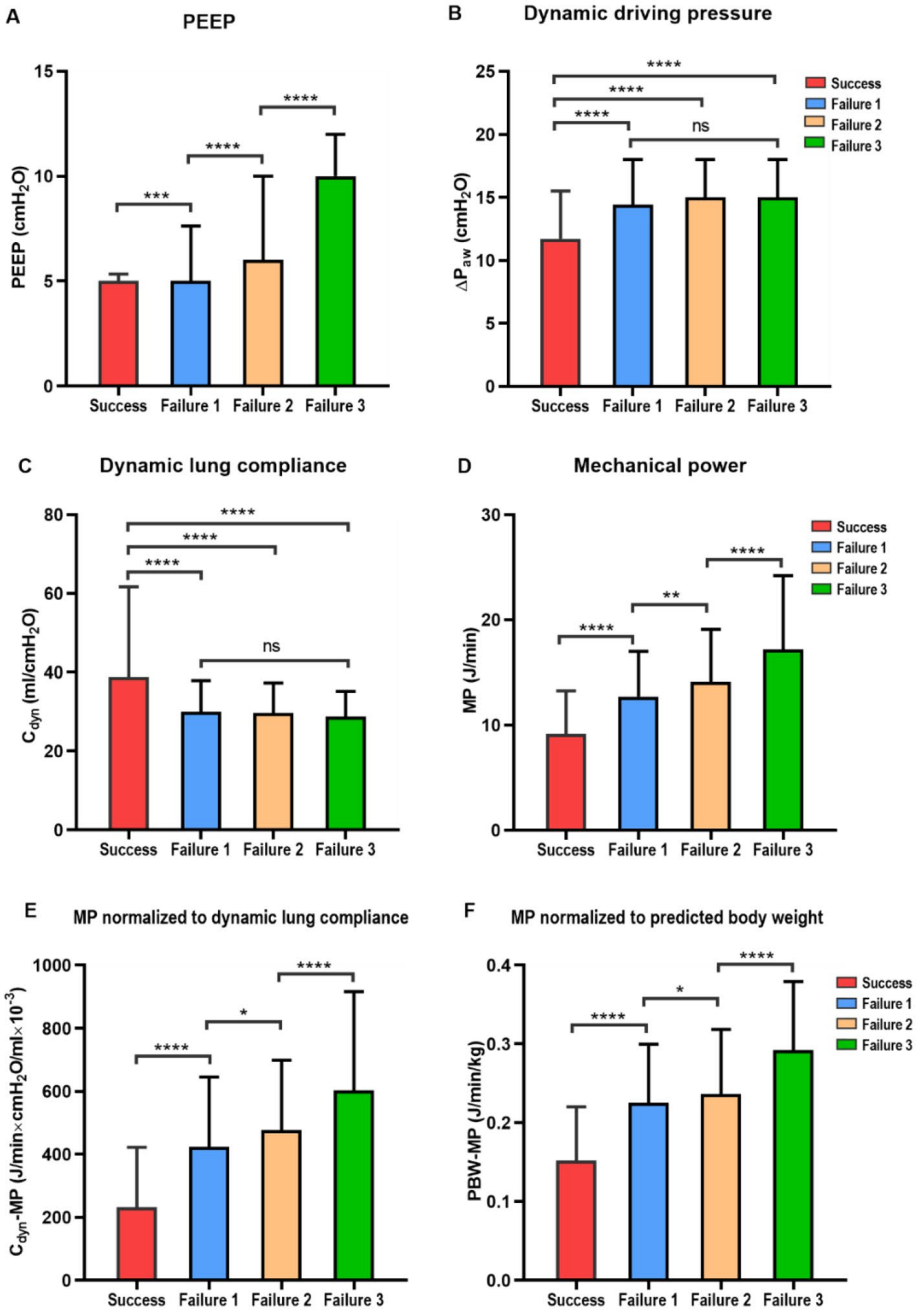
Comparison of respiratory mechanical parameters between weaning success and subgroups of weaning failure. (**A**) PEEP. (**B**) Dynamic driving pressure. (**C**) Dynamic lung compliance. (**D**) Mechanical power. (**E**) MP normalized to dynamic lung compliance. (**F**) MP normalized to predicted body weight (PBW). ****P < 0.0001; ***P < 0.001; **P < 0.01; *P < 0.05; ns, *P* > 0.05.

Significant differences between the weaning success group and the weaning failure group were detected in PEEP, MP, C_dyn_-MP, and PBW-MP (*P* < 0.001) (Fig. 3). Significant differences for the same variables were also detected between the weaning failure groups (*P* < 0.05). However, ∆P_aw_ and C_dyn_ were significantly different between the weaning success group and the weaning failure group (*P* < 0.0001), and there was no statistical difference between the groups of the weaning failure groups (Fig. 3). A comparison of the clinical characteristics of each group for weaning failure is shown in Supplementary Table S1.

**Figure 4:**
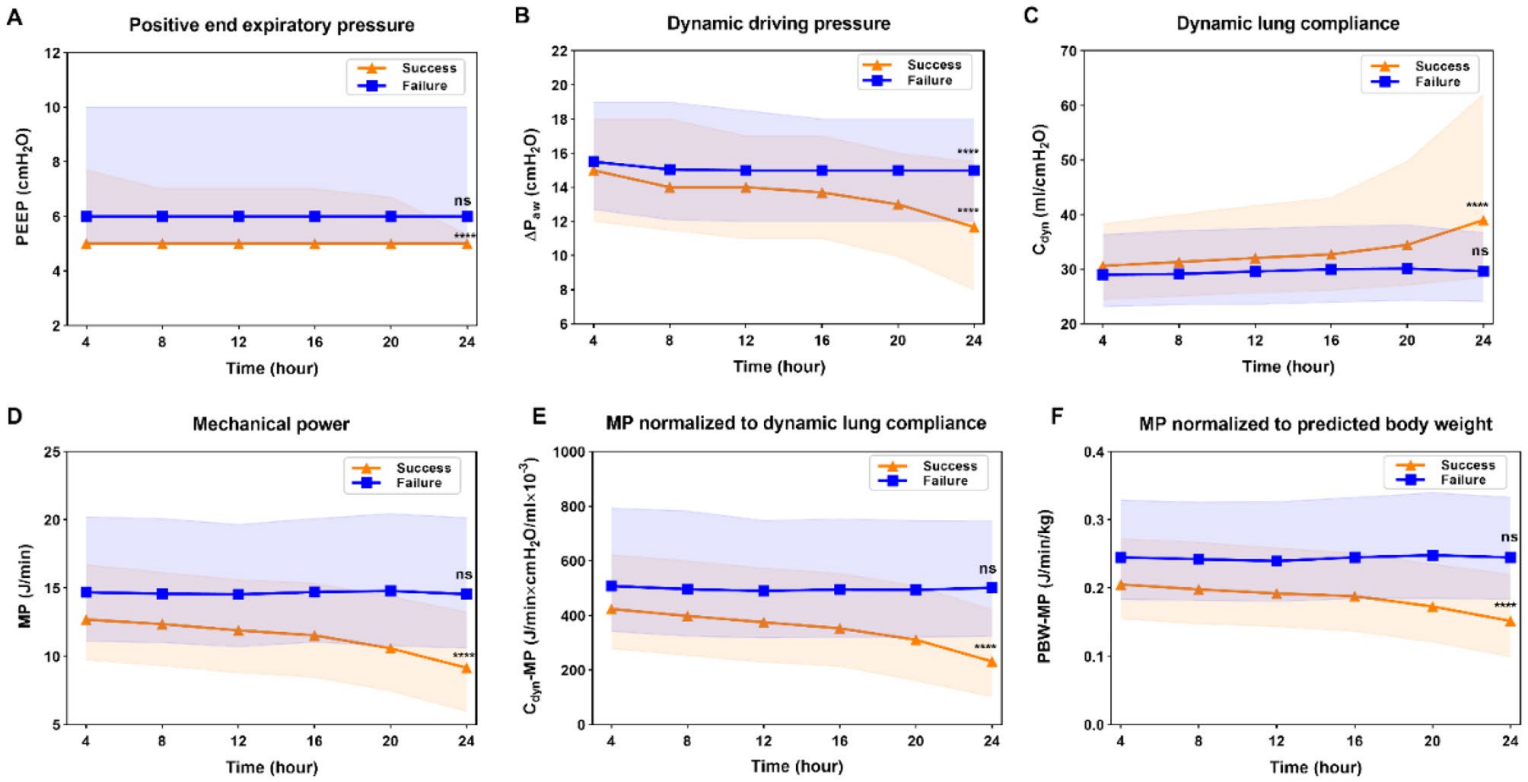
Comparison of dynamic respiratory mechanical parameters in weaning success and weaning failure group 24 h before SBT. (**A**) Positive and expiratory pressure. (**B**) Dynamic driving pressure. (**C**) Dynamic lung compliance. (**D**) Mechanical power. (**E**) MP normalized to dynamic lung compliance. (**F**) MP normalized to predicted body weight. ****P < 0.0001; ns, *P* > 0.05.

Compared with the first 4 h time frame, PEEP, ∆P_aw_, MP, C_dyn_-MP and PBW-MP of the last 4 h time frame within 24 h before SBT decreased significantly in the weaning success group, and C_dyn_ increased significantly compared with the first 4 h time frame. All were statistically significant (*P* < 0.0001) (Fig. 4). In the weaning failure group, except for ∆P_aw_, no statistical difference was detected in the dynamic comparison of respiratory mechanics indexes in each time frame (Fig. 4). Additionally, significant differences were detected in respiratory mechanics indexes between the successful weaning group and the weaning failure group in every 4 h time frame (*P* < 0.001) (Supplementary Table S3).

**Figure 5:**
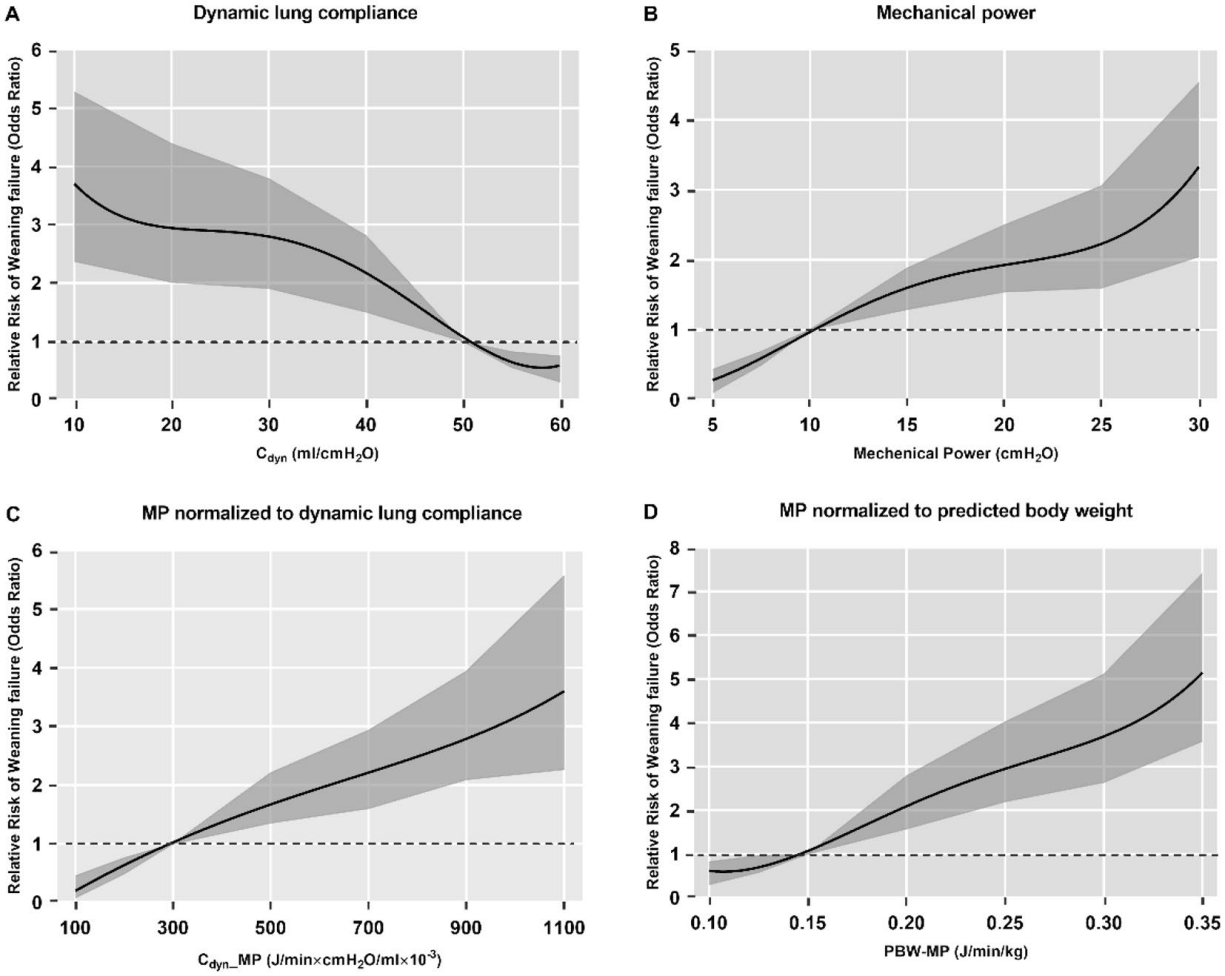
Modeled odds ratio for weaning failure according to dynamic lung compliance (C_dyn_), mechanical power (MP), MP normalized to dynamic lung compliance (C_dyn_-MP) and MP normalized to predicted body weight (PBW-MP) after multivariable adjustment for the covariates. The shaded areas represent the 95% confidence interval. (**A**) C_dyn_ adjusted for age, BMI, SOFA and MP and the mean VIF was 2.20. (**B**) MP adjusted for age, BMI, SOFA and C_dyn_ and the mean VIF was 1.98. (**C**) C_dyn_-MP adjusted for age, BMI, SOFA and C_dyn_ and the mean VIF was 1.66. (**D**) PBW-MP adjusted for age, BMI, SOFA and C_dyn_ and the mean VIF was 1.68. BMI, body mass index; SOFA, sequential organ failure assessment; VIF, variation inflation factor.

The variables C_dyn_, MP, C_dyn_-MP, PBW-MP were divided into six groups, with C_dyn_ (50–60 ml/cmH_2_O), MP (5–10 J/min), C_dyn_-MP (100–300 J/min × cmH_2_O/ml × 10^–3^), PBW-MP (0.10–0.15 J/min/kg) level used as the reference group, and logistic regression models were established for six subgroups of each variable. Figure 5 shows that decreasing C_dyn_ and increasing MP, C_dyn_-MP, and PBW-MP were all associated with an increased risk of weaning failure, after adjustment for confounders. Compared with the reference group, the OR value of weaning failure in the lowest C_dyn_ group increased significantly to 3.70 [95%CI (2.37–5.27), *P* < 0.001]. The OR values of weaning failure in the maximal MP, C_dyn_-MP, and PBW-MP groups increased to 3.33 [95%CI (2.04–4.53), *P* < 0.001], 3.58 [95%CI (2.27–5.56), *P* < 0.001], 5.15 [95%CI (3.58–7.41), *P* < 0.001], respectively (Fig. 5).

**Figure 6:**
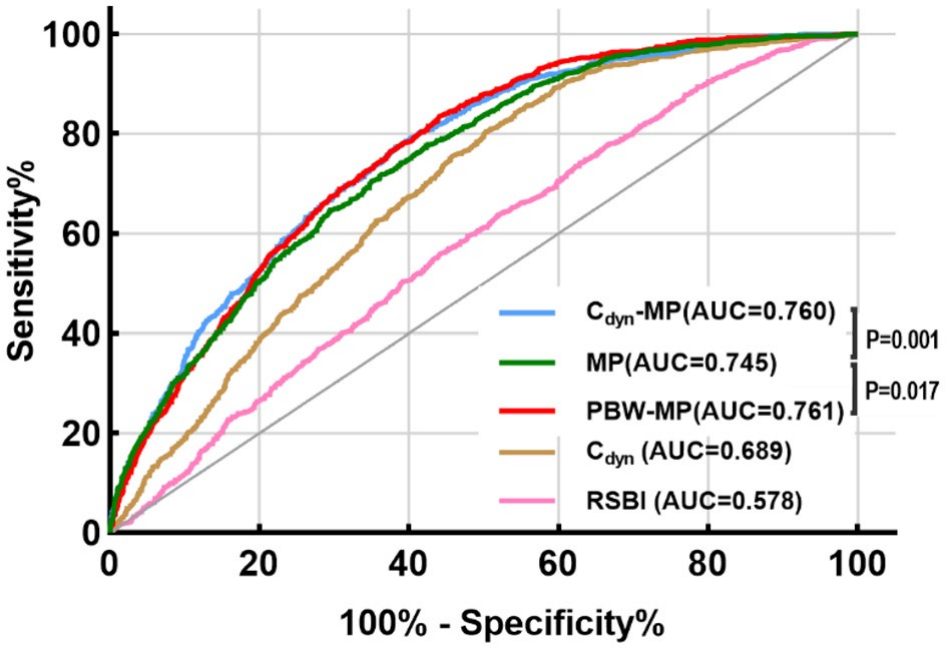
Comparison of receiver operating characteristic curves for RSBI, C_dyn_, MP, C_dyn_-MP, PBW-MP diagnosing weaning failure in all patients. RSBI: rapid shallow breathing index; C_dyn_: dynamic lung compliance; MP: mechanical power; C_dyn_-MP: MP normalized to dynamic lung compliance; PBW-MP: MP normalized to predicted body weight.

The AUROCs of RSBI, C_dyn_, MP, C_dyn_-MP, and PBW-MP for discriminating weaning failure were 0.578 [95%CI (0.560–0.597), *P* < 0.001], 0.689 [95%CI (0.672–0.706), *P* < 0.001], 0.745 [95%CI (0.730–0.761), *P* < 0.001], 0.760 [95%CI (0.745–0.776), *P* < 0.001] and 0.761 [95%CI (0.744–0.779), *P* < 0.001], respectively (Fig. 6). The discriminative performance of MP and norMP on the weaning outcome was significantly better than that of RSBI and C_dyn_, and the difference was statistically significant (*P* < 0.001). The discriminative performance of C_dyn_-MP and PBW-MP on the weaning outcome was stronger than that of MP, and the difference was statistically significant (*P* = 0.001, *P* = 0.017, respectively) (Fig. 6). Threshold ranges for MP, C_dyn_-MP, PBW-MP and other parameters for predicting weaning outcome were derived from ROC curves which is shown in Supplementary Table S4.

## Discussion

The results of this study showed that MP, MP normalized to dynamic lung compliance and MP normalized to PBW were independently associated with weaning outcomes in critically ill mechanically ventilated patients, and higher MP and norMP indicated an increased risk of weaning failure. In addition, the discriminative performance of norMP for weaning failure was better than that of MP. This is the first large-scale study to investigate the relationship between MP and weaning outcomes in mechanically ventilated patients based on the MIMIC-IV database.

MP is a unifying concept proposed by Gattinoni in the context of acute respiratory distress syndrome. MP integrates multiple factors of mechanical ventilation, and the total energy delivered by the ventilator to the lung parenchyma can be calculated from a combination of the following parameters tidal volume, PEEP, plateau pressure, peak inspiratory pressure, and respiratory rate^4,12^. The measurement of MP is simple and non-invasive, and the workload required to maintain optimal alveolar ventilation acting on the respiratory muscles per unit time can be obtained without disconnecting the ventilator at the bedside. Consequently, MP has also become a predictor to guide clinical weaning in recent years^5,6^. Studies have pointed out that MP is related to the size of functional lung volume, and MP standardized as a surrogate for lung size (such as well-inflated lung tissue determined by CT analysis, lung compliance, or predicted body weight) has higher diagnostic accuracy^4,13,14^. Therefore, while studying the correlation between MP and weaning outcomes, this paper also investigated whether norMP have higher discrimination ability for weaning failure than MP.

Among the 3695 mechanically ventilated patients in this study, 61.5% were successful in weaning after the first SBT, while 38.5% failed in weaning. The 28-day mortality rate of patients with weaning success was 12.4%, and the 28-day mortality rate of patients with weaning failure was 28.9%. A total of 11.1% of patients required reintubation 48 h after successful SBT weaning, which is consistent with the results of a multicenter observational study by Jaber et al.^15^. Compared with successful weaning patients, the duration of ventilation, length of stay in ICU, length of stay in hospital, 28-day reintubation rate, 28-day mortality were significantly longer in patients with weaning failure, while the 28-day ventilator-free days were significantly shortened, which is consistent with the findings of Hayashi et al.^16^. Multiple factors related to weaning failure were identified in the study, including disease severity (SAPS II, SOFA), sources of admission (SICU), respiratory load indicators (PEEP, P_plat_, P_peak_, ∆P_aw_, RR, MV, FiO_2_, MP, C_dyn_, C_dyn_-MP, PBW-MP), inflammatory markers (WBC), organ function and nutritional status (Albumiun, SCr), fluid management (Uorate), physiological status during weaning (HR, BF, MBP, SPO_2_, PH, PF), etc. (Table S2). The results showed that dynamic driving pressure (∆P_aw_) was associated with weaning outcomes, in contrast to driving pressure (∆P), which may be related to peak inspiratory pressure being a more conservative measure of elasticity than plateau pressure in nature^17^. Logistic regression analysis showed that MP, C_dyn_-MP, and PBW-MP were independently associated with weaning outcomes, which was consistent with the results of an observational study by Ghiani et al.^7^. This indicates that the pathophysiological mechanism of weaning failure is complex. Although the reversible factors leading to weaning failure have been actively treated, the imbalance between respiratory load and respiratory muscle performance remains the main reason for weaning failure^18^. MP includes multiple factors related to respiratory load, such as resistance, driving pressure, lung compliance and PEEP. A multitude of studies suggest that MP is more valuable for prognosis than a single parameter index^19,20^. The present study analyzed the respiratory mechanics indexes of ICU mechanically ventilated patients before weaning, and evaluated the relationship between MP, C_dyn_-MP, PBW-MP and weaning outcomes respectively, to further optimize the weaning decision. The results of this study showed that compared with the weaning success group, the C_dyn_ of the patients in the weaning failure group was lower, while the PEEP, ∆P_aw_, MP, C_dyn_-MP and PBW-MP were higher. Moreover, with the increasing difficulty of weaning, MP, C_dyn_-MP and PBW-MP increased significantly. Comparison of MP and norMP as comprehensive mechanical indicators with individual parameters such as C_dyn_ and ∆P_aw_, can not only reflect the weaning outcome of mechanically ventilated patients, but also reflect the severity of weaning difficulties.

The study also concluded that the dynamic comparison of respiratory mechanics indexes (including PEEP, C_dyn_, MP, C_dyn_-MP, and PBW-MP) 24 h before weaning in the weaning success group had significant differences, while there was no dynamic change in the weaning failure group. This shows that the dynamic reduction of respiratory load such as MP 24 h before weaning is a potent predictor of weaning success. In the process of mechanical ventilation, the power system of the ventilator overcomes the airway resistance of the patient and the elastic resistance of the respiratory system, thereby driving the gas into the alveoli to participate in the respiratory mechanical process of gas exchange^21,22^. Although PEEP can indicate the degree of stress inhomogeneity when recruiting lung tissue to a certain extent, it is only related to the static strain related to PEEP and cannot express the dynamic strain acting on the lung tissue during tidal ventilation^23^. In the majority of the cases, the level of ∆P_aw_ is simultaneously affected by the chest wall compliance and lung compliance of the patient. Simply using the driving pressure cannot accurately describe the compliance state of the lung^24^. MP integrates multiple respiratory mechanics parameters and can assess the impact of a mechanical breath on dynamic and static global lung strain and energy load^25^. In addition, based on ROC curve analysis, the discriminative performance of MP on the weaning outcome was stronger than that of traditional pre-weaning evaluation index RSBI and respiratory mechanics index C_dyn_. This shows that MP, which is a comprehensive respiratory mechanics index, can better reflect the workload exerted on the respiratory muscles before weaning.

The size of the lung volume that is ventilated determines the order of magnitude of energy per unit of lung tissue. Due to the different volume of the "baby lung", the different thoracic compliance, and the different ranges of strain and stress that the lung tissue can withstand in different regions, the conversion of MP normalized to ventilable lung volume may be necessary^9,13^. C_dyn_-MP, PBW-MP refer to the interaction between different lung pathophysiological features (including size, homogeneity and recruitability), reflecting the actual energy applied per unit of ventilated lung volume^4,26,27^. The results of this study showed that MP, C_dyn_-MP, and PBW-MP were all associated with an increased risk of weaning failure, higher MP or norMP values predicted greater risk of weaning failure. This indicates that the MP is related to the severity of lung disease before weaning and patients in the weaning failure group with "sicker" lungs require more intensive ventilation^28^. Several studies have linked MP > 12 J/min with the occurrence of ventilator-associated lung injury (VILI)^19,29^ and poor clinical prognosis^12,30–32^. Here, the MP cutoff value for predicting weaning failure was > 11.3 J/min (sensitivity 71%, specificity 65%), and the corresponding area under the curve was 0.745 (95% CI 0.730–0.761). The discriminative performance of C_dyn_-MP (AUROC 0.760 [95%CI 0.745–0.776]) and PBW-MP (AUROC 0.761 [95%CI 0.744–0.779]) was higher than that of MP (*P* < 0.05). This indicates that C_dyn_-MP and PBW-MP can better reflect the functional lung size and the pathophysiological state of the lungs during weaning in mechanical ventilation patients and are better representatives of the actual energy delivered by the ventilator to the lungs, thus having a higher predictive value for weaning outcomes.

There are some limitations to this study. First, we extracted data and calculated MP according to the simplified MP equation proposed by Gattinoni in the volume-controlled ventilation mode in our study. During our screening of the study population in the database, patients with missing all the required variables for the MP calculation were excluded, including all patients with missing P_plat_, that was, patients who did not have a P_plat_ measurement under volume-controlled ventilation before SBT. Because this study was a secondary analysis of the data set in MIMIC-IV for the purpose of clinical use, and there was no guarantee that the parameters were collected under standard conditions without spontaneous breathing and sufficient sedation level. Nevertheless, the average value of each parameter in every 4 h time frame in the present study was recorded for 24 h before weaning, and the dynamic changes of each respiratory mechanics index were monitored. Second, functional lung size was assessed primarily by lung compliance and predicted body weight, but computed tomography of the lung may be a more accurate method for the amount of remaining functional aerated lung, lung inhomogeneity, or recruitability estimation^9,33^. However, few data sets that include this kind of imaging exist at present, and further verification in large-scale clinical studies is needed. Finally, studies have shown that T-tube ventilation and pressure support strategies during spontaneous breathing trials may have an impact on weaning outcomes^10^. Therefore, only patients who used T-tube ventilation during weaning were included in this study, and further research on the predictive ability of MP in different SBT modes on the weaning outcome is needed.

## Conclusions

Mechanical power (MP) is associated with weaning outcomes in critically ill mechanically ventilated patients and independently predicts the risk of weaning failure. MP normalized to dynamic lung compliance and MP normalized to predicted body weight (PBW) produced better results than MP in discriminating weaning failure.

## Supplementary Information

Supplementary Tables.

## Data Availability

The datasets analysed during the current study are available in the MIMIC-IV repository, https://physionet.org/content/mimiciv/1.0/.
