# Supplementary material for: Mechanical power is associated with weaning outcome in critically ill mechanically ventilated patients
Source: Sci Rep. 2022 Nov 16;12:19634. doi: 10.1038/s41598-022-21609-2 (PMC9669041; doi:10.1038/s41598-022-21609-2)
Supplement: Supplementary file 1 — Supplementary Tables. [file 41598_2022_21609_MOESM1_ESM.docx]

**Mechanical power is associated with weaning outcome in critically ill mechanically ventilated patients**

Yao Yan, Yongpeng Xie, Xiaobing Chen, Yan Sun, Zhiqiang Du, Yanli Wang and Xiaomin Li

**Supplementary information**

**1. Glossary of terms (Page 2-3)**

**2. Table S1 Comparison of clinical characteristics among subgroups of**

**weaning failure (Page 4)**

**3. Table S2 Variables associated with weaning failure by binary logistic**

**regression (Page 5)**

**4. Table S3 Comparison of dynamic respiratory parameters before SBT**

**24h between weaning success and weaning failure (Page 6)**

**5. Table S4 Thresholds of variables used to predict the weaning outcome**

**by ROC curve Analysis (Page 7)**

|  |
| --- |

**6. References (Page 8)**

| **Glossary of terms** |  |
| --- | --- |
| **IMV** | Invasive mechanical ventilation |
| **MIMIC-IV** | Medical information mart for intensive care IV |
| **ICU** | Intensive care unit |
| **SBT** | Spontaneous breathing trial |
| **SQL** | Structured query language |
| **BMI** | Body mass index |
| **SAPS II** | Simplified acute physiology score II |
| **SOFA** | Sequential organ failure assessment |
| **MICU** | Medical intensive care unit |
| **SICU** | Surgical intensive care unit |
| **TSICU** | Trauma surgical intensive care unit |
| **MICU/SICU** | Medical/Surgical intensive care unit |
| **CVICU** | Cardiac vascular intensive care unit |
| **CCU** | Coronary care unit |
| **NSICU** | Neuro surgical intensive care unit |
| **COPD** | Chronic obstructive pulmonary disease |
| **WBC** | White blood cell count |
| **PLT** | Platelets |
| **Hb** | Hemoglobin |
| **SCr** | Serum creatinine |
| **Uorate** | Urine output rate |
| **HR** | Heart rate |
| **BF** | Breathing frequency |
| **MBP** | Mean blood pressure |
| **SPO_2_** | Pulse oximetry |
| **PF** | Arterial partial pressure of oxygen (PaO_2_) divided by the inspired  oxygen concentration (FiO_2_) |
| **LOS** | Length of stay |
| **V_T_** | Tidal volume |
| **RR** | Respiratory rate |
| **Positive end expiratory pressure (PEEP)** | Positive end-expiratory pressure (PEEP) as recorded was the external or applied PEEP, not the total PEEP, or intrinsic PEEP. |
| **Plateau pressure (P_plat_)** | The plateau pressure (P_plat_) was measured during an inspiratory pause on the ventilator. |
| **Peak inspiratory pressure (P_peak_)** | Peak inspiratory pressure (P_peak_) should be obtained while the patient is relaxed, not coughing or moving in bed. |
| **Dynamic driving pressure (ΔP_aw_)** | Dynamic driving pressure (ΔP_aw_) in the ventilation mode was calculated using P_peak_ and PEEP ^[1]^:   Δ**P_aw_ (cmH_2_O) = P_peak_ ‒ PEEP** |
| **Minute ventilation (MV)** | Minute ventilation (MV) was defined as the product of tidal volume (V_T_) and respiratory rate (RR). |
| **Driving pressure (ΔP)** | Driving pressure (ΔP) in the ventilation mode was calculated using P_plat_ and PEEP:   Δ**P (cmH_2_O) = P_plat_ ‒ PEEP** |
| **Mechanical power (MP)** | Mechanical power (MP) was calculated according to Gattinoni's simplified mechanical power equation as follows ^[2]^:   **MP (J/min) = 0.098×V_T_×RR×(P_peak_ – 0.5×ΔP)** |
| **Dynamic lung compliance (C_dyn_)** | Dynamic lung compliance (C_dyn_) refers to the change in lung volume caused by a unit pressure change, reflecting the compliance of the overall respiratory system ^[3]^ and was calculated using V_T_ and ΔP_aw_: **C_dyn_ = V_T_ /ΔP_aw_** |
| **MP normalized to dynamic lung compliance (C_dyn_-MP)** | MP normalized to dynamic lung compliance (C_dyn_-MP) was calculated using MP and C_dyn_ ^[4]^:   **C_dyn_-MP (J/min×cmH_2_O/ml×10^-3^) = MP /C_dyn_** |
| **MP normalized to predicted body weight (PBW-MP)** | MP normalized to predicted body weight (PBW-MP) was calculated using MP and predicted body weight (PBW) ^[5]^:   **PBW-MP (J/min/kg) = MP /PBW** Calculation of PBW ^[5]^:  **PBW (male) = 50 + 0.91 [height (cm) – 152.4]   PBW (female) = 45.5 + 0.91 [height (cm) – 152.4]** |
| **Rapid shallow breathing index (RSBI)** | Rapid shallow breathing index (RSBI) was calculated by dividing respiratory rate by tidal volume:   **RSBI = RR /V_T_** |

| **Variables** | **All** | **Group 1** | **Group 2** | **Group 3** | ***p* value** |
| --- | --- | --- | --- | --- | --- |
|  | **(n=1421)** | **(n=166)** | **(n=887)** | **(n=368)** |  |
| Age (years) | 66.5 (54.6-76.4) | 69.0 (59.4-78.0) | 65.9 (53.8-76.4) | 65.6 (54.7-75.5) | 0.059 |
| Gender (male) | 827 (58.2) | 97 (58.4) | 506 (57.1) | 224 (60.9) | 0.457 |
| BMI (kg/m^2^) | 28.5 (24.5-34.0) | 26.5 (23.9-31.8) | 28.4 (24.5-33.8) | 29.5 (25.5-35.9) | <0.001 |
| Smoking history | 128 (9.0) | 20 (12.1) | 83 (9.4) | 25 (6.8) | 0.122 |
| SAPS II | 44 (36-55) | 42 (34-53) | 44 (35-55) | 47 (38-59) | <0.001 |
| SOFA | 8 (5-11) | 7 (5-8) | 8 (5-11) | 9 (5-11) | <0.001 |
| **Sources of admission** |  |  |  |  |  |
| MICU | 325 (22.9) | 33 (19.9) | 218 (24.6) | 74 (20.1) | 0.142 |
| SICU | 205 (14.4) | 29 (17.5) | 117 (13.2) | 59 (16.0) | 0.211 |
| TSICU | 249 (17.5) | 38 (22.9) | 150 (16.9) | 61 (16.6) | 0.152 |
| MICU/SICU | 219 (15.4) | 14 (8.4) | 136 (15.3) | 69 (18.8) | 0.009 |
| CVICU | 198 (13.9) | 24 (14.5) | 121 (13.6) | 53 (14.4) | 0.919 |
| CCU | 143 (10.1) | 15 (9.0) | 95 (10.7) | 33 (9.0) | 0.579 |
| NSICU | 74 (5.2) | 13 (7.8) | 43 (4.8) | 18 (4.9) | 0.270 |
| Others | 8 (0.6) | 0 (0.0) | 7 (0.8) | 1 (0.3) | 0.315 |
| **Comorbidities** |  |  |  |  |  |
| Hypertension | 577 (40.6) | 70 (42.2) | 366 (41.3) | 141 (38.3) | 0.569 |
| Diabetes mellitus | 453 (31.9) | 51 (30.7) | 288 (32.5) | 114 (31.0) | 0.826 |
| COPD | 99 (7.0) | 15 (9.0) | 61 (6.9) | 23 (6.3) | 0.497 |
| Congestive heart failure | 432 (30.4) | 50 (30.1) | 262 (29.5) | 120 (32.6) | 0.558 |
| Coronary artery disease | 395 (27.8) | 45 (27.1) | 257 (29.0) | 93 (25.3) | 0.402 |
| Chronic kidney disease | 325 (22.9) | 37 (22.3) | 197 (22.2) | 91 (24.7) | 0.615 |
| Stroke | 294 (20.7) | 36 (21.7) | 178 (20.1) | 80 (21.7) | 0.757 |
| **Parameters before SBT 4h** | | | | | |
| V_T_ (ml) | 451 (398-507) | 461 (401-512) | 451 (400-508) | 449 (390-503) | 0.217 |
| PEEP (cmH_2_O) | 6.0 (5.0-10.0) | 5.0 (5.0-7.5) | 6.0 (5.0-10.0) | 10.0 (5.0-12.0) | <0.001 |
| P_plat_ (cmH_2_O) | 19.0 (16.0-22.0) | 18.0 (15.0-20.0) | 18.5 (15.5-22.0) | 21.0 (18.0-24.6) | <0.001 |
| P_peak_ (cmH_2_O) | 23.0 (19.0-27.0) | 21.0 (16.3-25.0) | 22.0 (18.0-26.0) | 25.0 (21.0-29.0) | <0.001 |
| ΔP_aw_ (cmH_2_O) | 15.0 (12.0-18.0) | 14.4 (11.0-18.0) | 15.0 (12.0-18.0) | 15.0 (12.0-18.0) | 0.378 |
| RR (bpm) | 20 (17-24) | 19 (16-23) | 20 (17-24) | 22 (18-26) | <0.001 |
| MV (l/min) | 8.8 (7.3-10.6) | 8.3 (7.0-9.9) | 8.7 (7.3-10.4) | 9.3 (7.9-11.3) | <0.001 |
| FiO_2_ (%) | 40 (40-50) | 40 (40-50) | 40 (40-50) | 50 (40-60) | <0.001 |
| MP (J/min) | 14.6 (10.6-20.2) | 12.7 (9.0-16.9) | 14.1 (10.4-19.1) | 17.2 (12.5-24.2) | <0.001 |
| C_dyn_ (ml/cmH_2_O) | 29.6 (24.2-36.7) | 30.0 (23.8-37.8) | 29.8 (24.3-37.3) | 28.8 (23.9-35.2) | 0.229 |
| C_dyn_-MP (J/min×  cmH_2_O) | 501.3 (323.4-744.8) | 424.8 (254.9-640.5) | 476.3 (314.9-698.5) | 603.5 (389.4-915.3) | <0.001 |
| PBW-MP | 0.2452 (0.1837-0.3331) | 0.2258 (0.1607-0.2982) | 0.2322 (0.1805-0.3151) | 0.2919 (0.2103-0.3794) | <0.001 |
| (J/min/kg) |  |  |  |  |  |
| RSBI (bpm/L) | 44.3 (35.1-56.5) | 40.0 (31.5-54.0) | 43.6 (35.0-55.0) | 46.7 (37.2-60.6) | <0.001 |
| **Laboratory data at the start of SBT** | |  |  |  |  |
| WBC (k/ul) | 11.9 (8.8-16.9) | 12.2 (9.6-17.3 ) | 11.8 (8.6-15.9) | 12.7 (9.0-18.8) | 0.049 |
| PLT (k/ul) | 164 (108-233) | 171 (127-252) | 165 (105-234) | 158 (103-223) | 0.043 |
| Hb (g/dl) | 10.0 (8.6-11.6) | 9.9 (8.9-11.4) | 9.9 (8.5-11.6) | 10.3 (8.8-11.8) | 0.157 |
| Albumiun (g/dl) | 2.9 (2.4-3.4) | 3.1 (2.7-3.5) | 2.9 (2.4-3.4) | 2.9 (2.3-3.3) | 0.004 |
| SCr (mg/dl) | 1.2 (0.8-2.0) | 1.1 (0.7-1.6) | 1.1 (0.8-2.0) | 1.3 (0.9-2.1) | 0.002 |
| Uorate before SBT (ml/kg/h) | 0.6 (0.3-1.1) | 0.6 (0.4-1.1) | 0.6 (0.3-1.1) | 0.5 (0.2-0.9) | <0.001 |
| **Physiological variables during SBT** | | | | | |
| HR (bpm) | 86 (73-99) | 85 (72-95) | 85 (72-97) | 88 (74-102) | 0.026 |
| BF (bpm) | 20 (17-24) | 18 (15-22) | 20 (17-24) | 21 (18-25) | <0.001 |
| MBP (mmHg) | 74 (67-83) | 76 (68-86) | 74 (67-83) | 73 (66-81) | 0.014 |
| SPO_2_ (%) | 98 (96-100) | 98 (96-100) | 98 (96 -100) | 98 (96-100) | 0.015 |
| Temperature (℃) | 37.1 (36.8-37.6) | 37.2 (36.9-37.6) | 37.1 (36.7-37.5) | 37.2 (36.8-37.6) | 0.084 |
| PH (mmHg) | 7.38 (7.33-7.43) | 7.38 (7.34-7.43) | 7.39 (7.34-7.44) | 7.37 (7.30-7.42) | <0.001 |
| PaO_2_ (mmHg) | 105 (83-129) | 107 (90-127) | 106 (84-132) | 101 (81-127) | 0.194 |
| PaCO_2_ (mmHg) | 39 (34 -45) | 39 (35-49) | 38 (33-44) | 40 (35-46) | 0.064 |
| PF (mmHg) | 226 (163-305) | 250 (188-330) | 236 (174-314) | 206 (133-268) | <0.001 |

**Table S1** Comparison of clinical characteristics among subgroups of weaning failure. Data are median (interquartile range) or no./total (%). BMI, body mass index; SAPS II, simplified acute physiology score II; SOFA, sequential organ failure assessment; MICU, Medical Intensive Care Unit; SICU, Surgical Intensive Care Unit; TSICU, Trauma SICU; MICU/SICU, Medical/Surgical Intensive Care Unit; CVICU, Cardiac Vascular Intensive Care Unit; CCU, Coronary Care Unit; NSICU, Neuro Surgical Intensive Care Unit; COPD, chronic obstructive pulmonary disease; SBT, spontaneous breathing trial; V_T_, tidal volume; PEEP, positive end expiratory pressure; P_plat_, plateau pressure; P_peak_, peak inspiratory pressure; ΔP_aw_, dynamic driving pressure (defined as P_peak_ – PEEP); RR, respiratory rate; MV, minute ventilation; MP, mechanical power; C_dyn_, dynamic lung compliance; C_dyn_-MP, MP normalized to dynamic lung compliance; PBW-MP, MP normalized to predicted body weight; RSBI, rapid shallow breathing index; WBC, white blood cell; PLT, platelet count; Hb, hemoglobin; SCr, serum creatinine; Uorate, Urine output rate; HR, heart rate; BF, breathing frequency; MBP, mean blood pressure; SPO_2_, pulse oximetry; PF, arterial partial pressure of oxygen (PaO_2_) divided by the inspired oxygen concentration (FiO_2_).

| **Variables** | **Univariable analysis** | | **Variables** | **Univariable analysis** | |
| --- | --- | --- | --- | --- | --- |
|  | **OR (95%CI)** | ***p* value** |  | **OR (95%CI)** | ***p* value** |
| Age | 1.00 (0.99-1.00) | 0.108 | PBW-MP, per 10^-2^ | 1.09 (1.08-1.10) | <0.001 |
| BMI | 1.00 (1.00-1.00) | 0.262 | J/min/kg |  |  |
| SAPS II | 1.01 (1.01-1.02) | <0.001 | WBC | 1.03 (1.02-1.04) | <0.001 |
| SOFA | 1.08 (1.06-1.10) | <0.001 | Albumiun | 0.84 (0.73-0.97) | 0.014 |
| SICU | 0.78 (0.65-094) | 0.008 | SCr | 1.10 (1.04-1.15) | <0.001 |
| PEEP | 1.33 (1.30-1.37) | <0.001 | Uorate | 0.85 (0.78-0.93) | <0.001 |
| P_plat_ | 1.14 (1.12-1.16) | <0.001 | HR | 1.01 (1.00-1.01) | <0.001 |
| P_peak_ | 1.16 (1.14-1.17) | <0.001 | BF | 1.08 (1.07-1.10) | <0.001 |
| ΔP_aw_ | 1.12 (1.10-1.14) | <0.001 | MBP | 0.99 (0.99-1.00) | <0.001 |
| RR | 1.09 (1.07-1.10) | <0.001 | SPO_2_ | 0.91 (0.89-0.94) | <0.001 |
| MV | 1.19 (1.15-1.22) | <0.001 | Temperature | 1.07 (0.96-1.20) | 0.214 |
| FiO_2_ | 1.05 (1.04-1.06) | <0.001 | PH, per 10^-1^ | 0.73 (0.64-0.84) | <0.001 |
| MP | 1.14 (1.13-1.16) | <0.001 | PF, per 10 mmHg | 0.97 (0.96-0.98) | <0.001 |
| C_dyn_ | 0.96 (0.96-0.97) | <0.001 |  |  |  |
| C_dyn_-MP, per 100 | 1.33 (1.29-1.36) | <0.001 |  |  |  |
| J/min×cmH_2_O/ml×10^-3^ |  |  |  |  |  |

**Table S2** Variables associated with weaning failure by binary logistic regression. BMI, body mass index; SAPS II, simplified acute physiology score II; SOFA, sequential organ failure assessment; SICU, Surgical Intensive Care Unit; PEEP, positive end expiratory pressure; P_plat_, plateau pressure; P_peak_, peak inspiratory pressure; ΔP_aw_, dynamic driving pressure (defined as P_peak_ – PEEP); RR, respiratory rate; MV, minute ventilation; MP, mechanical power; C_dyn_, dynamic lung compliance; C_dyn_-MP, MP normalized to dynamic lung compliance; PBW-MP, MP normalized to predicted body weight; WBC, white blood cell; SCr, serum creatinine; Uorate, Urine output rate; HR, heart rate; BF, breathing frequency; MBP, mean blood pressure; SPO_2_, pulse oximetry; PF, arterial partial pressure of oxygen (PaO_2_) divided by the inspired oxygen concentration (FiO_2_).

| **Parameters before SBT 24h** | | **4h** | **8h** | **12h** | **16h** | **20h** | **24h** | ***p* value** |  |
| --- | --- | --- | --- | --- | --- | --- | --- | --- | --- |
|  |  |  |  |  |  |  |  |  |  |
| V_T_ (ml) | Weaning success | 455 (402-508) | 456 (402-512) | 453 (401-511) | 453 (400-511) | 454 (397-514) | 452 (392-519) | 0.809 |  |
|  | Weanging failure | 453 (400-511) | 454 (401-512) | 454 (402-511) | 454 (401-511) | 453 (400-507) | 451 (398-507) | 0.555 |  |
| PEEP (cmH_2_O) | Weaning success | 5.0 (5.0-7.7)^a^ | 5.0 (5.0-7.0)^a^ | 5.0 (5.0-7.0)^a^ | 5.0 (5.0-7.0)^a^ | 5.0 (5.0-6.7)^a^ | 5.0 (5.0-5.3)^a,b^ | <0.001 |  |
|  | Weanging failure | 6.0 (5.0-10.0) | 6.0 (5.0-10.0) | 6.0 (5.0-10.0) | 6.0 (5.0-10.0) | 6.0 (5.0-10.0) | 6.0 (5.0-10.0) | 0.167 |  |
| P_plat_ (cmH_2_O) | Weaning success | 17.0 (15.0-20.0)^a^ | 17.0 (14.5-20.0)^a^ | 17.0 (14.5-20.0)^a^ | 17.0 (14.5-20.0)^a^ | 17.0 (14.0-20.0)^a^ | 17.0 (14.0-20.0)^a,b^ | <0.001 |  |
|  | Weanging failure | 19.0 (16.0-23.0) | 19.0 (16.0-23.0) | 19.0 (16.0-23.0) | 19.0 (16.0-22.5) | 19.0 (16.0-22.7) | 19.0 (16.0-22.0) | 0.760 |  |
| P_peak_ (cmH_2_O) | Weaning success | 21.0 (18.0-25.0)^a^ | 20.5 (17.0-24.0)^a^ | 20.0 (17.0-24.0)^a^ | 20.0 (16.0-24.0)^a^ | 19.0 (15.0-23.0)^a^ | 17.0 (12.8-21.0)^a,b^ | <0.001 |  |
|  | Weanging failure | 23.0 (19.0-27.5) | 23.0 (19.0-27.0) | 23.0 (19.0-27.0) | 23.0 (19.0-27.0) | 23.0 (19.0-27.0) | 23.0 (19.0-27.0) | 0.080 |  |
| ΔP_aw_ (cmH_2_O) | Weaning success | 15.0 (12.0-18.0)^a^ | 14.0 (11.5-18.0)^a^ | 14.0 (11.0-17.0)^a^ | 13.7 (11.0-17.0)^a^ | 13.0 (10.0-16.0)^a^ | 11.7 (8.0-15.5)^a,b^ | <0.001 |  |
|  | Weanging failure | 15.5 (12.7-19.0) | 15.1 (12.1-19.0) | 15.0 (12.0-18.5) | 15.0 (12.0-18.0) | 15.0 (12.0-18.0) | 15.0 (12.0-18.0) | <0.001 |  |
| RR (bpm) | Weaning success | 18 (16-22)^a^ | 18 (16-22)^a^ | 18 (16-22)^a^ | 18 (16-22)^a^ | 18 (16-21)^a^ | 18 (16-22)^a,b^ | <0.001 |  |
|  | Weanging failure | 20 (17-24) | 20 (17-24) | 20 (16-24) | 20 (17-24) | 20 (17-24) | 20 (17-24) | 0.812 |  |
| MV (l/min) | Weaning success | 8.1 (7.0-9.6)^a^ | 8.1 (7.0-9.6)^a^ | 8.1 (6.9-9.5)^a^ | 8.0 (6.9-9.5)^a^ | 8.0 (6.8-9.4)^a^ | 7.9 (6.7-9.5)^a,b^ | <0.001 |  |
|  | Weanging failure | 8.7 (7.4-10.6) | 8.8 (7.4-10.7) | 8.7 (7.3-10.5) | 8.8 (7.4-10.6) | 8.8 (7.4-10.7) | 8.8 (7.3-10.6) | 0.958 |  |
| MP (J/min) | Weaning success | 12.7 (9.7-16.7)^a^ | 12.4 (9.3-16.1)^a^ | 11.9 (8.8-15.6)^a^ | 11.6 (8.5-15.4)^a^ | 10.6 (7.5-14.4)^a^ | 9.2 (6.0-13.2)^a,b^ | <0.001 |  |
|  | Weanging failure | 14.7 (11.1-20.2) | 14.6 (11.0-20.1) | 14.6 (10.7-19.7) | 14.7 (11.1-20.1) | 14.8 (10.8-20.5) | 14.6 (10.6-20.2) | 0.680 |  |
| C_dyn (_ml/cmH_2_O) | Weaning success | 30.6 (24.5-38.3)^a^ | 31.3 (25.1-40.0)^a^ | 32.1 (25.7-41.7)^a^ | 32.7 (26.2-43.1)^a^ | 34.5 (27.1-49.8)^a^ | 39.0 (28.5-62.0)^a,b^ | <0.001 |  |
|  | Weanging failure | 29.0 (23.2-36.3) | 29.1 (23.5-37.1) | 29.6 (23.6-37.4) | 30.0 (24.0-37.9) | 30.1 (24.3-38.1) | 29.6 (24.2-36.7) | 0.128 |  |
| C_dyn_-MP (J/min×  cmH_2_O/ml×10^-3^) | Weaning success | 423.4 (278.5-622.0)^a^ | 397.7 (254.0-599.2)^a^ | 374.9 (228.9-572.9)^a^ | 352.8 (214.1-553.8)^a^ | 310.5 (161.2-508.0)^a^ | 231.5 (101.7-421.6)^a,b^ | <0.001 |  |
|  | Weanging failure | 508.0 (341.8-792.8) | 496.1(324.0-782.7) | 489.3 (318.5-747.4) | 495.6 (320.8-752.6) | 493.9 (320.8-747.2) | 501.3 (323.4-744.8) | 0.386 |  |
| PBW-MP (J/min/kg) | Weaning success | 0.2053  (0.1554-0.2723)^a^ | 0.1983 (0.1478-0.2668)^a^ | 0.1923 (0.1436-0.2585)^a^ | 0.1883 (0.1367-0.2517)^a^ | 0.1730 (0.1212-0.2352)^a^ | 0.1520 (0.0995-0.2200)^a,b^ | <0.001 |  |
|  | Weanging failure | 0.2451  (0.1836-0.3291) | 0.2423 (0.1824-0.3258) | 0.2398 (0.1811-0.3266) | 0.2452 (0.1846-0.3328) | 0.2483 (0.1849-0.3398) | 0.2452 (0.1837-0.3331) | 0.710 |  |

**Table S3** Comparison of dynamic respiratory parameters before SBT 24h between weaning success and weaning failure. Data are median (interquartile range) or no./total (%). SBT, spontaneous breathing trial; V_T_, tidal volume; PEEP, positive end expiratory pressure; P_plat_, plateau pressure; P_peak_, peak inspiratory pressure; ΔP_aw_, dynamic driving pressure (defined as P_peak_ – PEEP); RR, respiratory rate; MV, minute ventilation; MP, mechanical power; C_dyn_, dynamic lung compliance; C_dyn_-MP, MP normalized to dynamic lung compliance; PBW-MP, MP normalized to predicted body weight; ^a^, *P*<0.001 weaning success vs weaning failure; ^b^, *P*<0.001 24h vs 4h within group comparison.

| **Variables** | **AUC (95%CI)** | ***P* value** | **Thresholds** | **Sensitivity** | **Specificity** | **PPV** | **NPV** |
| --- | --- | --- | --- | --- | --- | --- | --- |
| RR (bpm) | 0.619 (0.600-0.637) | <0.001 | >19.8 | 0.58 | 0.58 | 0.69 | 0.47 |
| PEEP (cmH_2_O) | 0.712 (0.695-0.729) | <0.001 | >5.4 | 0.59 | 0.75 | 0.74 | 0.60 |
| P_plat_ (cmH_2_O) | 0.645 (0.627-0.664) | <0.001 | >18.3 | 0.54 | 0.68 | 0.70 | 0.51 |
| ΔP_aw_ (cmH_2_O) | 0.667 (0.650-0.684) | <0.001 | >12.7 | 0.70 | 0.57 | 0.75 | 0.50 |
| C_dyn_ (ml/cmH_2_O) | 0.689 (0.672-0.706) | <0.001 | <31.3 | 0.57 | 0.67 | 0.71 | 0.52 |
| FiO_2_ (%) | 0.625 (0.606-0.644) | <0.001 | >42.9 | 0.51 | 0.69 | 0.69 | 0.51 |
| PF (mmHg) | 0.580 (0.552-0.608) | <0.001 | <257.3 | 0.39 | 0.52 | 0.59 | 0.50 |
| MP (J/min) | 0.745 (0.730-0.761) | <0.001 | >11.3 | 0.71 | 0.65 | 0.56 | 0.78 |
| C_dyn_-MP (J/min×cmH_2_O/ml×10^-3^) | 0.760 (0.745-0.776) | <0.001 | >317.8 | 0.76 | 0.63 | 0.56 | 0.81 |
| PBW-MP (J/min/kg) | 0.761 (0.744-0.779) | <0.001 | >0.1796 | 0.77 | 0.62 | 0.57 | 0.81 |
| **Table S4** Thresholds of variables used to predict the weaning outcome by receiver operating characteristic curve analysis. RR, respiratory rate; PEEP, positive end expiratory pressure; P_plat_, plateau pressure; ΔP_aw_, dynamic driving pressure; C_dyn_, dynamic lung compliance; PF, arterial partial pressure of oxygen (PaO_2_) divided by the inspired oxygen concentration (FiO_2_); MP, mechanical power; C_dyn_-MP, MP normalized to dynamic lung ompliance; PBW-MP, MP normalized to predicted body weigh. | | | | | | | |

**References**

1. Chiu LC, Lin SW, Chuang LP, et al. Mechanical power during extracorporeal membrane oxygenation and hospital mortality in patients with acute respiratory distress syndrome. Crit Care. **25**(1), 13 (2021).
2. Gattinoni L, Tonetti T, Cressoni M, et al. Ventilator-related causes of lung injury: the mechanical power. Intensive Care Med. **42**(10),1567–1575 (2016).
3. Ghiani A, Paderewska J, Walcher S, et al. Mechanical power normalized to lung-thorax compliance indicates weaning readiness in prolonged ventilated patients. Sci Rep.**12**(1), 6 (2022).
4. Baptistella AR, Mantelli LM, Matte L, et al. [Prediction of extubation outcome in mechanically ventilated patients: Development and validation of the Extubation Predictive Score (ExPreS).](https://pubmed.ncbi.nlm.nih.gov/33735250/) Plos one. **16**(3), e0248868 (2021).
5. Zhang ZH, Zheng B, Liu N, et al. Mechanical power normalized to predicted body weight as a predictor of mortality in patients with acute respiratory distress syndrome. Intensive Care Med. **45**(6), 856–864 (2019).
